# Brachytherapy with Iodine-125 seeds for treatment of portal vein-branch tumor thrombus in patients with hepatocellular carcinoma

**DOI:** 10.1186/s12885-021-08680-0

**Published:** 2021-09-14

**Authors:** Duo Hong, Yi Zhou, Xiaoting Wan, Hongying Su, Haibo Shao

**Affiliations:** 1 Department of Interventional Radiology, The First Hospital of China Medical University, No.155 Nanjing Road, Heping District, Shenyang, 110000 Liaoning China; 2 Vascular Surgery and Interventional Department, Changzhou No.2 People’s Hospital, 29 Xinglong Lane, Tianning District, Changzhou, 213000 Jiangsu China; 3 Department of Nuclear Medicine, Sun Yat-sen Memorial Hospital, Sun Yat-sen University, Guangzhou, 510000 China

**Keywords:** Hepatocellular carcinoma, Portal vein branch tumor thrombosis, Brachytherapy, Iodine-125 seeds

## Abstract

**Background:**

There is currently no widely-accepted consensus for the management of hepatocellular carcinoma with portal vein tumor thrombus. We evaluate the safety and efficacy of ultrasound-guided percutaneous brachytherapy with iodine-125 seeds for the treatment of hepatocellular carcinoma with portal vein-branch tumor thrombus (PVBTT).

**Methods:**

Sixty-nine hepatocellular carcinoma patients with PVBTT were enrolled; 34 received transarterial chemoembolization (TACE) combined with iodine-125 seeds implanted in the PVBTT; 35 were treated with TACE alone. Adverse events, objective response rate, disease control rate, progression-free survival, and overall survival were compared between the two groups. Tumor responses of PVBTT and intrahepatic tumor were correlated. Multivariate and subgroup analyses were conducted for overall survival.

**Results:**

No grade 3 or 4 adverse events were recorded, and there was no difference in grade 1 or 2 adverse events between the two groups. Objective response rate and disease control rate for PVBTT were 58.9 and 91.2%, respectively, in the combined treatment group, which were significantly greater than the 5.7 and 54.3% rates, respectively, in the TACE-alone group (both *p*’s ≤ 0.001). Intrahepatic tumor response was positively correlated with the PVBTT response (γ = 0.782, *p* < 0.01). Survival outcomes were better in the combined treatment group than in the TACE-alone group: the median progression-free survival for PVBTT was 9 months versus 3 months (HR = 0.187 [95% CI: 0.101, 0.345], *p* < 0.001), and the median overall survival was 11 months versus 7 months (HR = 0.448 [95% CI: 0.265, 0.758], *p* = 0.003). Multivariate analysis revealed that application of brachytherapy and lower grade PVBTT (Vp1 + Vp2 vs. Vp3) were protective predictors of overall survival. In stratified analysis, the benefit of overall survival was more significant in the subgroup of PVBTT Vp1 + Vp2 rather than in Vp3.

**Conclusions:**

The combination of iodine-125 seed brachytherapy guided by ultrasound and TACE is a convenient, safe, and effective treatment for patients with HCC and PVBTT, conferring a better survival benefit than TACE alone.

**Supplementary Information:**

The online version contains supplementary material available at 10.1186/s12885-021-08680-0.

## Background

Hepatocellular carcinoma (HCC) is the sixth most common cancer and the third most prevalent cause of cancer-related death worldwide [1]. HCC has a propensity to invade adjacent vasculature, and portal vein tumor thrombus (PVTT) is present in about 10–40% of HCC patients at the time of diagnosis [2]. PVTT is aggressive and generally develops from the branch to the main trunk in a short amount of time, leading to portal vein hypertension, hematemesis from the rupture of collateral vessels, ascites, and ischemic liver damage [3]; median overall survival (OS) is 2.7 to 4 months in this circumstance [4]. According to the Barcelona Clinic Liver Cancer staging system, tyrosine kinase inhibitors, including sorafenib and lenvatinib, are the standard treatment for HCC patients with PVTT (stage C) [5], which has extended the median OS only to 6.5 months [6]. Therefore, other therapeutic modalities to improve the survival of such patients are being tried, including surgical resection, transarterial chemoembolization (TACE), chemotherapy, and three-dimensional conformal radiotherapy, but the clinical outcomes remain unsatisfactory. Recently, stent placement plus brachytherapy with iodine-125 seeds has been reported in the treatment of HCC with main portal vein tumor thrombus, with a median OS of 9.3 months [7]. In our center, ultrasound-guided percutaneous brachytherapy with iodine-125 seeds has been performed for portal vein branch tumor thrombus (PVBTT), which refers to portal vein thrombi that have not reached the main trunk. The administration of brachytherapy for PVBTT is aimed at preventing progression of tumor thrombi into the main trunk, especially for patients who cannot physically or economically afford tyrosine kinase inhibitors. Ultrasound-guided brachytherapy can be delivered in one session, with a higher local dose of radiation than with conventional radiotherapy. Therefore, we have conducted a retrospective study to evaluate the safety and effectiveness of ultrasound-guided percutaneous brachytherapy for the treatment of HCC with PVBTT.

## Methods

### Patients

In this retrospective study, we reviewed the records of 235 patients collected from the institutional database of interventional radiology from the last 5 years (2015–2019) who had HCC complicated with PVTT. The diagnosis of HCC was based on the American Association for the Study of Liver Diseases practice guidelines on the management of HCC [8]. PVTT was confirmed by the enhancement of an expanding intraluminal mass in the portal vein on the arterial phase, and a low-attenuation intraluminal filling defect on the portal phase of contrast-enhanced computed tomography (CT) or dynamic magnetic resonance imaging (MRI) [9]. The inclusion criteria for entry into the study were (a) age between 18 and 75 years; (b) Barcelona Clinic Liver Cancer C stage; (c) Eastern Cooperative Oncology Group (ECOG) performance status of 0–2; (d) Child-Pugh class A or B; and (e) Vp1–3, according to Japan’s VP staging classification system [10]; Vp1, presence of a tumor thrombus distal to, but not within, the second-order branches; Vp2, presence of a tumor thrombus in the second-order branches; Vp3, presence of a tumor thrombus in the first-order branches. The exclusion criteria were (a) previous treatment for the tumor, including surgery, radiotherapy, TACE, molecular targeting therapy, and immunotherapy, and (b) Vp4, presence of a tumor thrombus in the main trunk of the portal vein. Sorafenib was primarily recommended for the patients with HCC with PVBTT. If the patients declined sorafenib, the combination TACE and iodine-125 brachytherapy was recommended, but some patients were unable to receive implantation because the visuality of lesions through ultrasound was affected by gastrointestinal or pulmonary gas. Some patients rejected iodine-125 implantation. TACE alone was performed for these patients. The final choices were made by the patients. No bias advice was given by doctors. According to these criteria, 69 patients were included and divided into two groups. Patients in the TACE-iodine-125 group received combined treatments of iodine-125 seed implantation and TACE; patients in the TACE group were treated with TACE alone. This clinical study was approved by the Ethics Committee of the First Hospital of China Medical University, and written informed consent was obtained from all patients before the procedures.

### Treatments

**Fig. 1:**
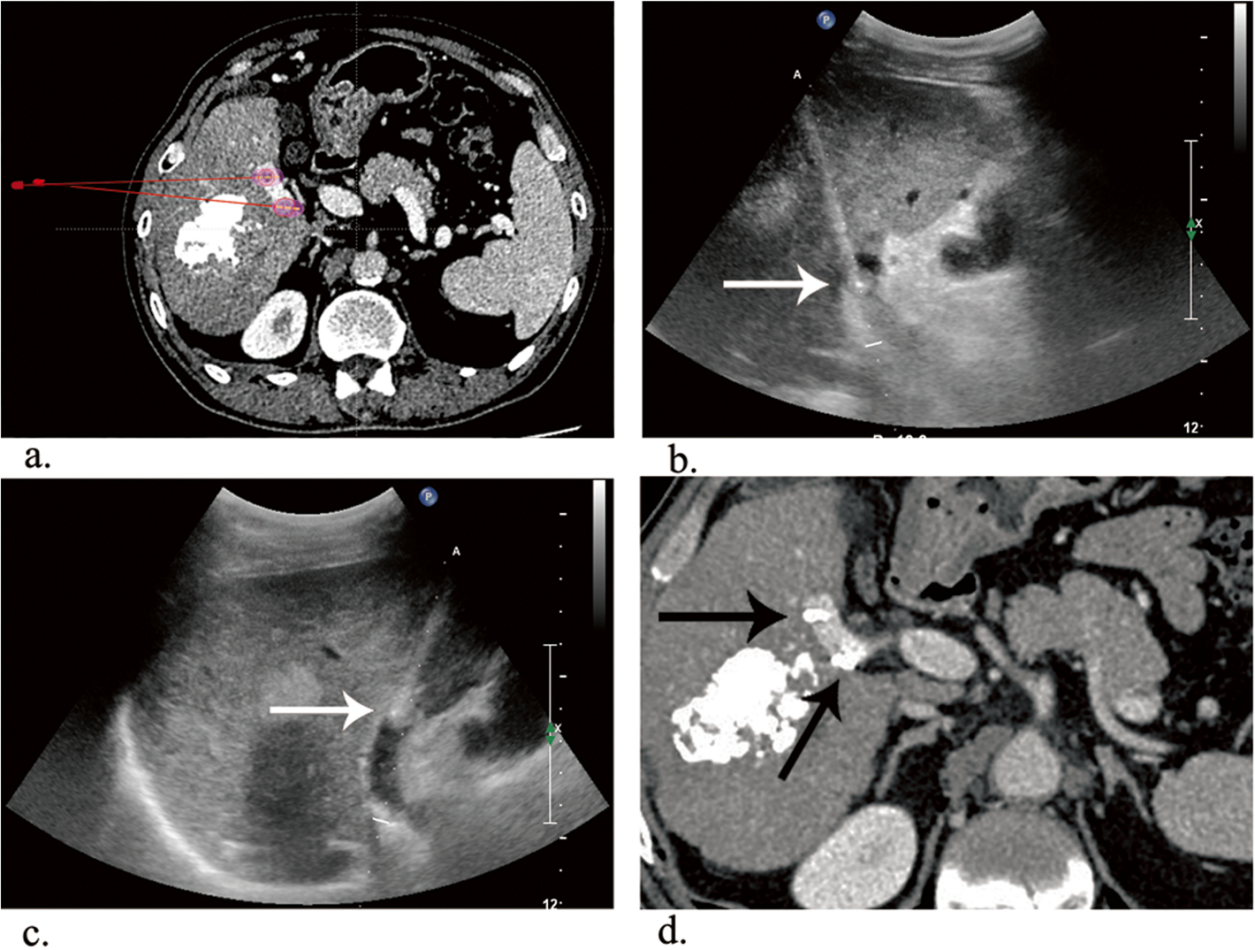
**a** Contrast-enhanced CT of two 1-cm diameter tumor thrombi floating in the right segment of the portal vein. The preprocedural Treatment Planning System (TPS) was applied. **b** The needle (white arrow) punctured the proximal thrombus with ultrasound guidance. **c** The needle (white arrow) was placed in the distal thrombus. **d** Contrast-enhanced CT at the 1-month follow-up. Tumor response was evaluated as CR (black arrows)

Because of physical or economic constraints, the enrolled patients did not receive any systemic treatments. Both groups received conventional lipiodol TACE [11]. - Hepatic arteriography was conducted to detect feeding arteries of the HCC and existence of arterioportal shunts (APS). If APS was confirmed, the location, severity and direction of the vessels was identified through further arteriography, then super selection was performed with a microcatheter (Progreat, Terumo, Japan) advancing into the feeding artery of APS. Microspheres with a diameter of 300–900 μm (Embosphere, Merit Medical, USA) were injected to block the APS. Arteriography was then performed to confirm the occlusion of APS. Lastly, a microcatheter was inserted into the feeding arteries of the HCC to deliver 40 mg epirubicin (Pharmorubicin, Pfizer, USA) mixed with 4–20 mL (mean 15.4 ± 5.3 mL) iodized oil (Youliying, Hengrui, China) under fluoroscopic guidance. Brachytherapy was performed for the TACE-Iodine-125 group 1–2 weeks after the administration of TACE. The procedure was performed under ultrasound guidance (Philips, Royal Dutch Philips, Netherlands) with an in-plane ultrasound needle guide (Ultra-Pro, CIVCO, Iowa, USA). The iodine-125 seed (Beijing Atomic Technology Company, China) was 4.5 mm in length and 0.8 mm in diameter. The radioactivity of each seed was 0.7 mCi, with a half-life of 59.6 days. The radiation energy was 27.4 KeV, with an effective irradiation range of 17 mm. The prescription dose of radiation was set to 120 Gy. Preprocedural contrast-enhanced images were imported into the treatment planning system (Beijing University of Aeronautics and Astronautics, China) to evaluate the number and locations of seeds and access paths of the needles. According to the designed paths, 18 G Chiba needles were directed into the PVBTT under ultrasound guidance, and the seeds were placed by an implantation device (Fig. 1). CT examination was performed immediately after the procedures, and the images were input to the treatment planning system again to verify the doses of the implantations.

### Follow-up

Follow-up was conducted for all patients, with contrast-enhanced CT or MRI 1 month after completion of the initial treatments, and follow-up intervals of 2–3 months thereafter. TACE was repeated if there was radiologic confirmation of residual or recurrent intrahepatic tumor.

### Adverse events evaluation

Adverse events were graded according to the Common Toxicity Criteria for Adverse Events version 3.0 [12]. Image examinations (lung and liver) in each follow-up visit and DSA imaging in each TACE procedure were used to identify if seed migration occurred. Radiation-induced liver disease (RILD) was categorized into classic and nonclassic types. Classic RILD was defined as anicteric hepatomegaly and ascites with more than double the upper limit of the normal level of alkaline phosphate (AKP); nonclassic RILD was defined as ≥ grade 3 hepatic toxicities, with more than 3 times the upper limit of the normal level of blood bilirubin or more than 5 times the upper limit of the normal levels of ALT or AST [13]. In order to avoid interference of initial TACE, RILD was evaluated at 1 month and 4 months after implantation (first and second follow-up visits, respectively).

### Therapeutic efficacy evaluation

Intrahepatic tumor response was evaluated between baseline and best response (the greatest shrinkage of intrahepatic tumor after treatments on the follow up images) according to the modified Response Evaluation Criteria in Solid Tumors (mRECIST) [14]. PVBTT response was evaluated between baseline and best response by a modified standard: the product of the largest perpendicular diameters of the tumor thrombus was calculated and compared to the initial value [15]. The response of the PVBTT was defined as complete response (CR), complete disappearance of the PVBTT; partial response (PR), ≥50% decrease in the value; stable disease (SD), < 50% decrease or < 25% increase; and progressive disease (PD), ≥25% increase. The objective response rate (ORR) was based on the combined number of patients with CR and PR; the disease control rate (DCR) included patients with CR, PR, and SD. Progression-free survival (PFS) was defined as the period from the day of the procedure until radiologic confirmation of tumor progression or death. PVBTT PFS was calculated separately according to the progressive disease of the tumor thrombus. Overall survival (OS) was defined as the period from initial treatment to death or last visit.

### Statistical analysis

All statistical analyses were performed with SPSS 20 software (SPSS, IBM, USA) and R software (version 3.6.2, http://www.Rproject.org). For analyses of the baseline characteristics, continuous variables are presented as the mean ± SD and were compared by Student’s t-test or Mann-Whitney U test. Categorical variables are expressed as frequencies and were compared using the χ2 test. The ORR and DCR between the two groups were compared with the χ2 test. Pearson correlation analysis was conducted to validate the correlation between the PVBTT response (percentage change of product) and intrahepatic tumor response (percentage change of tumor diameter). The Kaplan-Meier method was used to estimate PFS and OS curves, and the log-rank test was used to compare the curves between groups. All univariate and multivariate analyses were performed using Cox proportional hazards models. Only variables with *p* < 0.1 in the univariate analyses were entered into the multivariate analyses. Backward elimination Cox regression was applied to select the risk factors in the multivariate analyses. All statistical tests were two tailed, and a *p* value less than 0.05 indicated a significant difference.

## Results

Among the 69 patients included in the study, 34 were in the TACE-iodine-125 group, and 35 were in the TACE group. The mean age of the entire cohort was 56.2 ± 8.0 years. The baseline characteristics of the TACE-iodine-125 group and the TACE group were not significantly different except for a slightly higher AFP value in the TACE-only patients (*p* = 0.032) (Table 1). TACE was performed 2.5 ± 0.9 (range 1–6) times. An average of 15 iodine-125 seeds were implanted in the patients in the combined treatment group. The duration of the procedure was 10–20 min (mean 15.2 ± 4.7 min). All the iodine-125 brachytherapy and TACE procedures were technically successful.

**Table 1:** Baseline characteristics of the patients

Variable	TACE-Iodine-125 (*n* = 34) (%)	TACE (*n* = 35) (%)	*p* Value
Sex			0.163†
Male	29(85)	25(71)	
Female	5(15)	10(29)	
Age (y) (mean ± SD)	58.1 ± 7.3	54.5 ± 8.4	0.061‡
ECOG performance			0.729†
0	15(44)	14(40)	
1–2	19(56)	21(60)	
Cause of liver disease			
HBV	31(91.2)	33(94.4)	0.973§
HCV	2(5.9)	1(2.8)	0.980§
Other	1(2.9)	1(2.8)	
Child-Pugh score			0.627§
A	33(97)	32(91.4)	
B	1(3)	3(8.6)	
Largest tumor diameter (cm) (mean ± SD)	7.6 ± 3.0	8.7 ± 2.5	0.119‡
Number of tumors			0.956†
Single	17(45.9)	20(54.1)	
Multiple	18(56.2)	14(43.8)	
Extrahepatic spread	3(9)	4(11)	1.000§
AFP level (ng/mL)			0.032†*
≤ 400	15(44)	7(20)	
> 400	19(56)	28(80)	
ALT (u/l) (Mean ± SD)	42.9 ± 23.6	46.5 ± 30.3	0.584§§
TBIL (μmol/L) (Mean ± SD)	16.4 ± 7.8	20.1 ± 10.1	0.067§§
Albumin (g/L) (Mean ± SD)	37.2 ± 4.6	34.7 ± 6.2	0.114§§
Type of PVTT			0.722†
VP1 + VP2	17(50)	16(46)	
VP3	17(50)	19(54)	

*HBV* hepatitis B virus, *HCV* hepatitis C virus, *AFP* alpha-fetoprotein, *ALB* albumin, *ALT* alanine transaminase, *TBIL* total bilirubin

* *p* < 0.05

† Pearson χ2 test

‡ Independent-samples *t* test

§ Continuity corrected

§§ Mann-Whitney U test

Adverse events related to the procedures are listed in Table 2. Grades 1 and 2 post-chemoembolization syndrome (fever, vomiting, and abdominal pain) occurred in 27 patients (79.4%) in the TACE-iodine-125 group and 30 patients (85.7%) in the TACE group (*p* = 0.49). The patients tolerated these symptoms with general care, and no further invasive management was required. No complications related to seed implantation, such as hemorrhage, implantation metastasis and seed migration, occurred. According to evaluation criteria, no RILD was found at 1 month and 4 months after implantation. The laboratory examination results for RILD are shown in Supplemental Fig. 1.

**Table 2:** Adverse events

Adverse events	TACE-	AEs Grade			TACE	AEs Grade			*p*
	I-125	1	2	≥3		1	2	≥3	
Fever	25/34	17	8	0	29/35	20	9	0	0.348†
Nausea or vomiting	11/34	9	2	0	10/35	5	5	0	0.733†
Abdominal pain	16/34	13	3	0	12/35	8	4	0	0.280†
Hemorrhage	0/34	0	0	0	0/35	0	0	0	–
Seed migration	0/34	0	0	0	–	–	–	–	–
RILD	0/34	0	0	0	–	–	–	–	–

*RILD* radiation-induced liver disease

† Pearson χ2 test

**Fig. 2:**
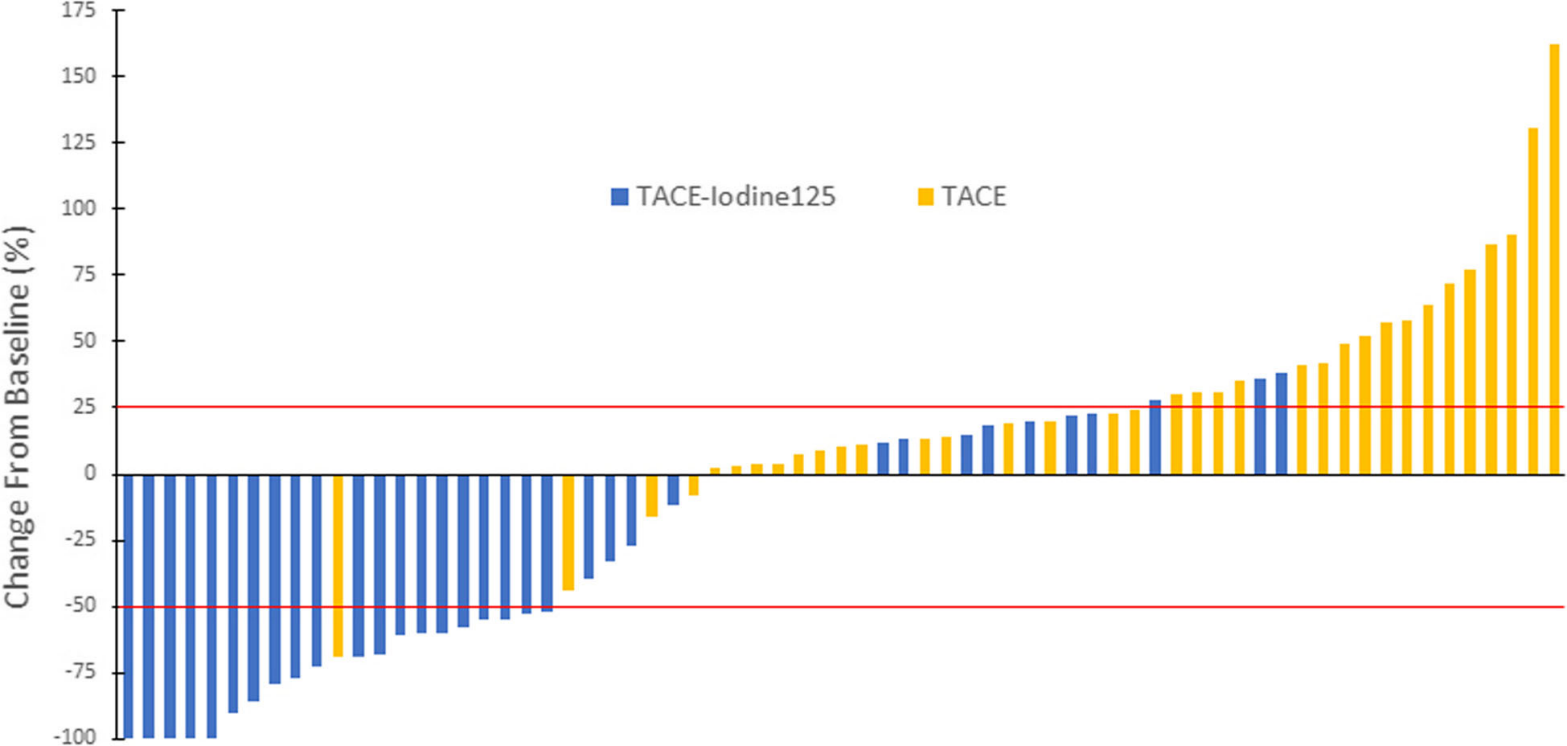
PVBTT response rate in the TACE-Iodine-125 group (blue bars) and the TACE group (yellow bars). The PVBTT response rate is presented as change in the product of perpendicular diameters of the tumor thrombosis from baseline

**Fig. 3:**
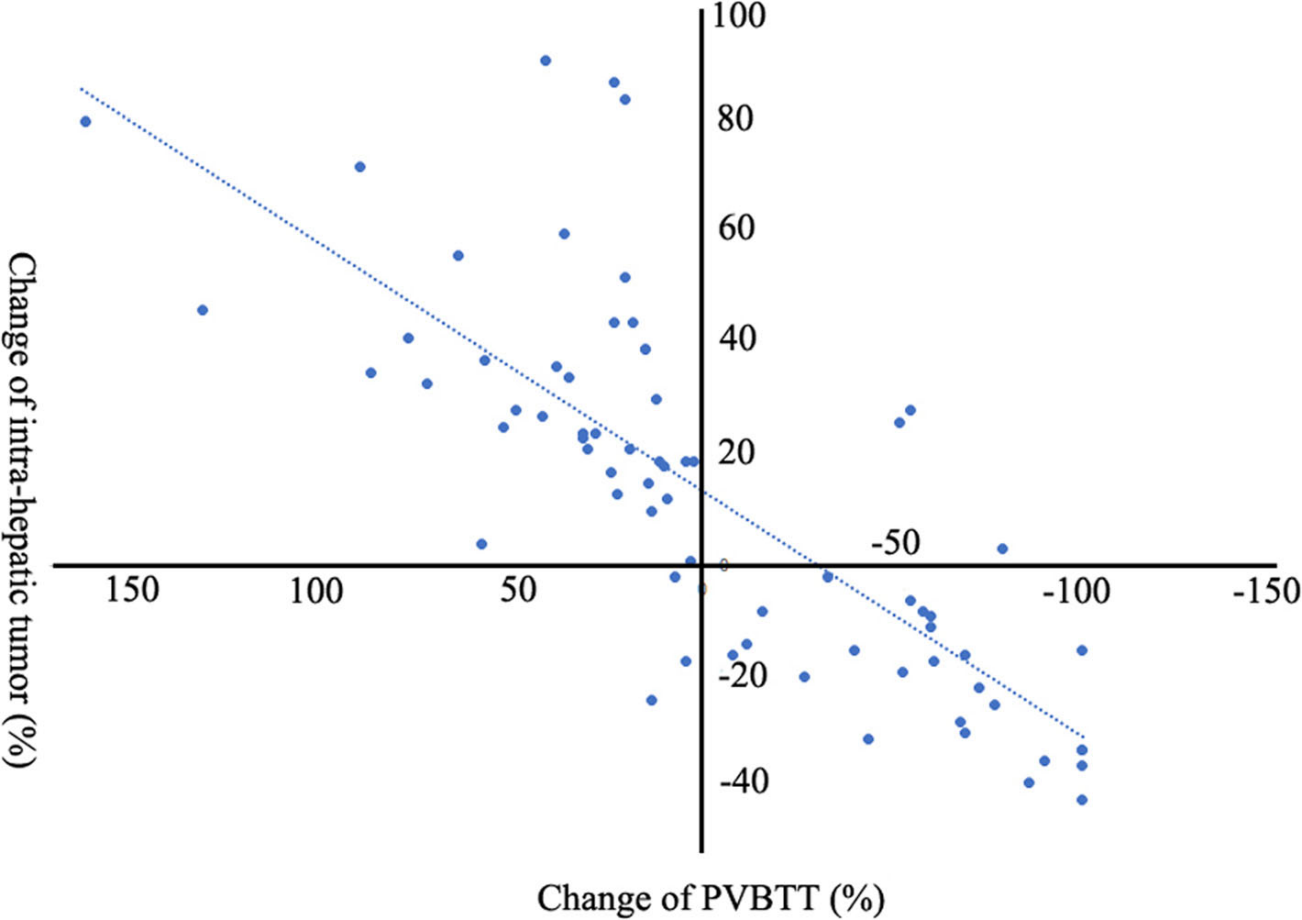
Scatter plot of the PVBTT and intrahepatic tumor response rate in the entire cohort. The intrahepatic tumor response rate was positively correlated with the PVBTT response rate (γ = 0.782, *p* < 0.01)

Tumor responses of the intrahepatic tumor and PVBTT are given in Table 3. The ORR and DCR for PVBTT were significantly higher in the TACE-iodine-125 group than in the TACE group (58.9% vs. 5.7, 91.2% vs. 54.3%; both p’s < 0.001). Similarly, the DCR for intrahepatic tumor was significantly greater in the TACE-iodine-125 group than in the TACE group (73.5% vs. 48.6%, *p* = 0.034). Figure 2 illustrates the individual PVBTT response rate from baseline to best response. For the entire cohort, Pearson correlation analysis indicated that the intrahepatic tumor response rate was positively correlated with the PVBTT response rate (γ = 0.782, *p* < 0.01) (Fig. 3).

**Table 3:** Intrahepatic tumor and PVBTT responses in the two groups

Tumor Response	Intrahepatic Tumor			PVBTT		
	TACE-I-125	TACE	*p*	TACE-I-125	TACE	*p*
Complete response (CR)	0	0		5	0	
Partial response (PR)	6	2		15	2	
Stable disease (SD)	19	15		11	17	
Progressive disease (PD)	9	18		3	16	
Objective response rate (ORR) (%)	17.6	5.7	0.188‡	58.9	5.7	< 0.001†*
Disease control rate (DCR) (%)	73.5	48.6	0.034†*	91.2	54.3	0.001†*

* *p* < 0.05

† Pearson χ2 test was used

‡ Continuity correction was used

**Fig. 4:**
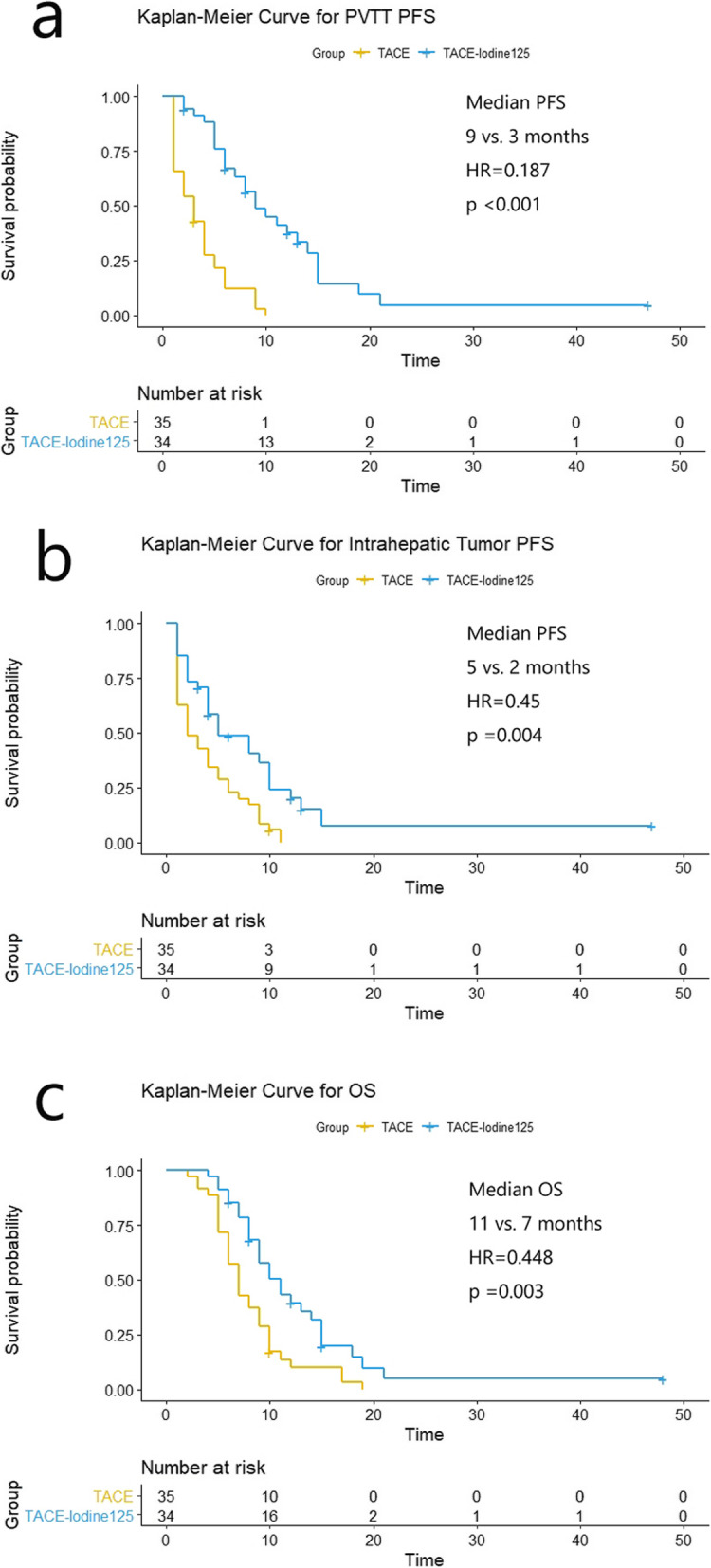
Kaplan-Meier curves. **a** Comparison of PVBTT PFS survival curves. **b** Comparison of intrahepatic tumor PFS curves. **c** Comparison of the OS curves

The median follow-up period was 8 months (range 2–48 months). The patients in the TACE-iodine-125 group had a significantly longer median PFS for PVBTT than did those in the TACE group (9 vs. 3 months, HR = 0.187 [95% CI: 0.101, 0.345, *p* < 0.001]) (Fig. 4a). In addition, the median PFS for intrahepatic tumors was significantly longer in the TACE-iodine-125 group than in the TACE group (5 vs. 2 months, HR = 0.450 [95% CI: 0.263, 0.769, *p* = 0.004]) (Fig. 4b). The patients receiving TACE iodine-125 treatment had a median OS of 11 months, whereas those who received TACE alone had a median OS of 7 months (HR = 0.448 [95% CI: 0.265, 0.758, *p* = 0.003]) (Fig. 4c). Furthermore, OS was compared between the patients whose PVBTT responses were CR + PR and those with SD + PD. The median OS was 13 months in the CR + PR group and 7 months in the SD + PD group (HR = 0.323 [95% CI: 0.176, 0.589, *p* < 0.001]).

Univariate and multivariate analyses were further conducted for OS. As shown in Table 4, the factors of treatment modality, type of PVTT, largest tumor diameter and AFP achieved a *p* value < 0.1 in the univariate analysis and thus entered the multivariate analysis. The multivariate Cox proportional hazard model revealed that treatment modality (TACE- iodine − 125 vs. TACE, HR = 0.482 [95% CI: 0.276, 0.841, *p* = 0.01]) and type of PVTT (Vp1 + Vp2 vs. Vp3, HR = 0.399 [95% CI: 0.229, 0.696, *p* = 0.001]) were independent predictors of OS.

**Table 4:** Univariate and multivariate analyses for OS

Risk Factor	Univariate			Multivariate		
	HR	95% CI	*p* Value	HR	95% CI	*p* Value
Sex (F vs. M)	1.452	0.804–2.621	0.216			
Age (≤60 y vs. > 60 y)	1.039	0.597–1.807	0.893			
ECOG (0 + 1 vs. 2)	0.840	0.335–2.110	0.711			
Child (A vs. B)	0.583	0.209–1.629	0.304			
Treatment method (TACE-I-125 vs. TACE)	0.448	0.265–0.758	0.003**	0.482	0.276 ~ 0.841	0.010*
Type of PVTT (VP1 + VP2 vs. VP3)	0.421	0.247–0.719	0.002**	0.399	0.229 ~ 0.696	0.001*
Number of tumors (Single vs. Multiple)	0.800	0.479–1.338	0.352			
Tumor Size (≤5 vs. > 5)	0.699	0.362–1.348	0.285			
Extrahepatic metastasis (No vs Yes)	0.933	0.423–2.063	0.863			
AFP (≤400 ng/mL vs. > 400 ng/mL)	0.606	0.344–1.066	0.082**	0.706	0.388 ~ 1.285	0.254

* *p* < 0.05

**Fig. 5:**
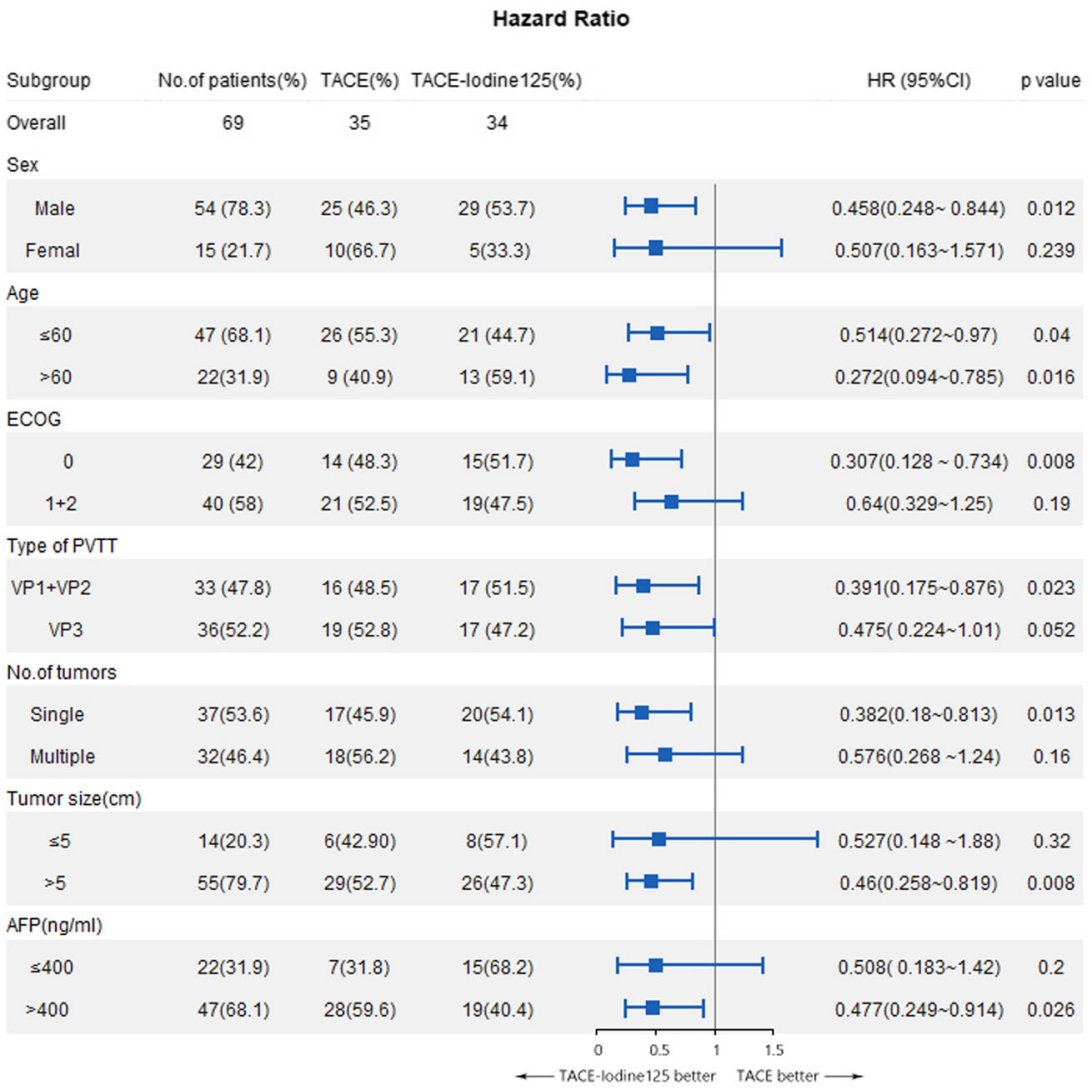
Subgroup analysis of overall survival hazard ratios based on treatment modality

For the two treatment modalities, the HRs of OS were determined in subgroups (Fig. 5). Treatment with TACE-iodine-125 provided a better OS in the subgroups of male, ECOG = 0, type of Vp1 + Vp2, single tumor, size > 5 cm, and AFP > 400 ng/mL, compared OS of the subgroups of female, ECOG = 1 + 2, type of VP3, multiple tumors, size ≤5 cm, and AFP ≤ 400 ng/mL. The median OS for PVBTT types was further analyzed: In the Vp1 + Vp2 subgroup, the median OS was significantly longer in the TACE-iodine-125 group than in the TACE-alone group (13 vs. 7.5 months, HR = 0.391 [95% CI: 0.175, 0.876, *p* = 0.017], whereas in the Vp3 subgroup, the median OS was not significantly different for the two treatment modalities (8 vs. 7 months, HR = 0.475 [95% CI: 0.224–1.01, *p* = 0.052)].

## Discussion

There is currently no widely-accepted consensus for the management of HCC with PVTT [16]. The main role of TACE is to control intrahepatic lesions, but the effectiveness of controlling PVTT is not satisfactory. Our study revealed that combined treatment of TACE and implantation iodine-125 seeds had a better tumor response and significant survival benefit over TACE alone in the patients with PVBTT. In the subgroup analysis, this survival benefit was confirmed for the patients who were male, of any age, ECOG = 0, VP1 + VP2 thrombus, single tumor, tumor size > 5 cm, or AFP > 400 ng/mL, Thus, iodine-125 implantation in PVBTT was an effective supplementary method to TACE in treatment of patients with advanced HCC.

The presence of PVTT is considered a strongly negative prognostic factor in HCC patients due to increasing risk of intrahepatic tumor dissemination through arterioportal shunt. Yang et al. (15) retrospectively reviewed 379 HCC patients who underwent TACE as initial treatment and found that the patients with lipiodol deposition in the PVTT had a longer OS than did those without lipiodol deposition [17]. Our study has thus demonstrated, with correlation analysis, that effective treatment of PVBTT could improve intrahepatic tumor control and accordingly prolong survival time. The median OS was better in the patients whose PVBTT responded (CR + PR, 13 months) rather than in the patients without response (SD + PD, 7 months). Therefore, control of the PVTT is as important as treatment of intrahepatic tumors for patients with advanced HCC.

In our study, PVTT classification was the only independent predictor of OS aside from treatment modality. The survival benefit of local brachytherapy of PVBTT in the Vp3 subgroup was not statistically significant, with a median survival time of 8 vs. 7 months (*p* = 0.052). Some studies have reported similar results with other treatment modalities [18, 19]. Lee et al. (16) reported that the median OS of patients with Vp1 and Vp2 thrombus were significantly different between patients who did or did not undergo liver resection, TACE, or sorafenib administration (*p* = 0.003); however, for Vp3 PVTT, such a difference was not significant (*p* = 0.499). The 1-year survival rate in Vp3 patients was only half that of Vp1 + Vp2 patients (35% vs. 71.4%) [18]. Two possible reasons for this survival difference are 1) the number of branches involved increased exponentially as the PVTT grade rose, but the radiation range remained limited, and 2) Vp3 PVTT often has a greater intrahepatic tumor burden than Vp1 and Vp2 [20] {Ahn, 2020 #428}. Therefore, PVTT should be detected and treated earlier to obtain better long-term outcomes.

CT guidance was used in most previous studies of iodine-125 treatment of PVTT [21, 22]. However, the non–real-time procedures in this study were guided by ultrasound. Unlike with CT guidance, the direction of the needle can be adjusted precisely in real time with ultrasound, thereby substantially reducing operation time and complications. The ultrasound guiding approach has disadvantages, however, as the images it acquires cannot be imported into the treatment planning system immediately to verify effective distribution after seed implantation, and examination of the lesion is sometimes affected by gastrointestinal and lung gas.

At present, liver resection (LR) and liver transplantation (LT) are proposed increasingly for HCC with vascular invasion because of recent advances in surgical techniques and perioperative management; however, OS differ significantly according to the extent and severity of PVTT. The combined use of surgery and multimodal therapies could improve long-term survival. Chiang et al. combined TACE and SBRT as a preoperative therapy for BCLC stage B and C HCC with appropriate patient selection and the results were encouraging: the ORR was 68%; the 1-year LC rate was 93.6%; and the median OS was 19.8 months [23]. We think the capability of iodine-125 seed brachytherapy is similar to that of SBRT; it could improve survival after curative therapies for advanced HCC.

The distribution of AFP ≤ 400 and > 400 patients varies between the two groups (*p* = 0.032) in baseline characteristics, which could impact outcomes. Elevated AFP levels have been reported to be associated with poor OS in HCC patients with PVTT [24], but there were not enough samples for further matching to balance all clinical factors, and AFP was not an independent predictor in univariate or multivariate analyses for OS in similar brachytherapy studies that included AFP ≤ 400 and > 400 into baseline characteristics in the last 5 years [21, 22, 25, 26].

Our study has limitations. 1) It is retrospective, and the sample size was small. 2) Some patients were enrolled to the TACE group because the detection of their lesions through ultrasound was affected by gastrointestinal or pulmonary gas. One of the reasons for the lack of detection was hepatatrophy caused by cirrhosis, which may affect liver function and survival. 3) Targeted agents and immune checkpoint inhibitors have been approved for systemic therapy of advanced HCC, but they were not considered in our study, which results in a relatively limited application. Multicenter randomized controlled trials with a large cohort of patients treated with brachytherapy and immune checkpoint inhibitors may be warranted in the future.

## Conclusion

The combination of iodine-125 seed brachytherapy guided by ultrasound and TACE is a convenient, safe, and effective treatment for patients with HCC and PVBTT, conferring a better survival benefit than TACE alone.

## Supplementary Information

**Additional file 1: Supplemental Fig. 1.** Details of laboratory examination for RILD. (a) TBIL test before implantation, at 1 month and 4 months after implantation. The threshold value was 3 times the upper limit of the normal level (20 μmol/L). (b) AKP test before implantation, at 1 month and 4 months after implantation. The threshold value was 2 times the upper limit of the normal level (125 U/L). (c) ALT test before implantation, at 1 month and 4 months after implantation. The threshold value was 5 times the upper limit of the normal level (50 U/L). (d) AST test before implantation, at 1 month and 4 months after implantation. The threshold value was 5 times the upper limit of the normal level (40 U/L).

## Data Availability

The datasets used and analyzed during the current study are available from the corresponding authors on reasonable request.

## Abbreviations

AFP: Alpha-fetoprotein
DCR: Disease control rate
ECOG: Eastern Cooperative Oncology Group
HCC: Hepatocellular carcinoma
HR: Hazard ratio
mRECIST: Modified Response Evaluation Criteria in Solid Tumors
ORR: Objective response rate
OS: Overall survival
PD: Progressive disease
PFS: Progression-free survival
PR: Partial response
PVBTT: Portal vein-branch tumor thrombus
PVTT: Portal vein tumor thrombus
SD: Stable disease
TACE: Transarterial chemoembolization
