# Supplementary figures and images for: Brachytherapy with Iodine-125 seeds for treatment of portal vein-branch tumor thrombus in patients with hepatocellular carcinoma
Source: BMC Cancer. 2021 Sep 14;21:1020. doi: 10.1186/s12885-021-08680-0 (PMC8439081; doi:10.1186/s12885-021-08680-0)

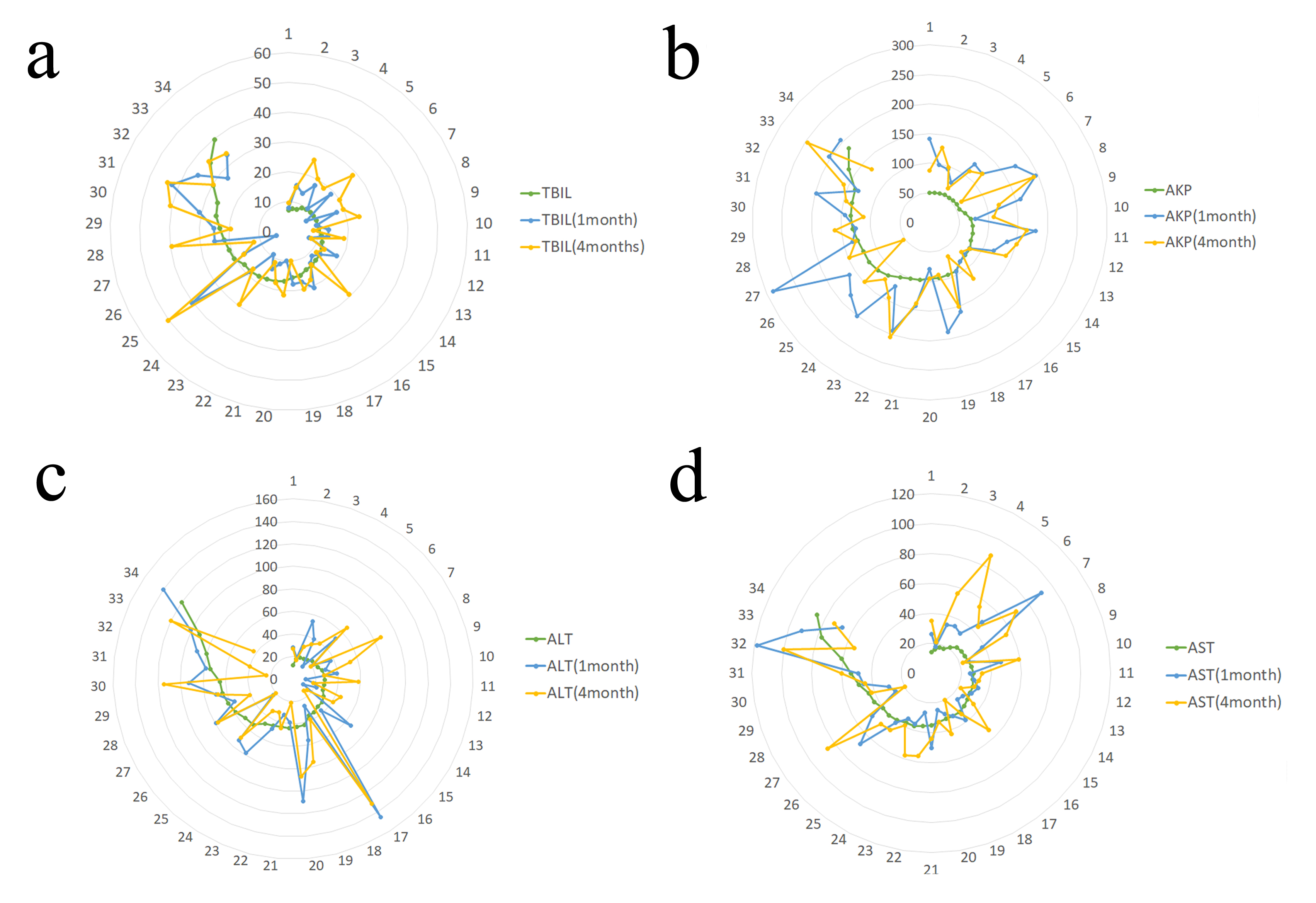

Supplement: Supplementary file 1 — Additional file 1: Supplemental Fig. 1. Details of laboratory examination for RILD. (a) TBIL test before implantation, at 1 month and 4 months after implantation. The threshold value was 3 times the upper limit of the normal level (20 μmol/L). (b) AKP test before implantation, at 1 month and 4 months after implantation. The threshold value was 2 times the upper limit of the normal level (125 U/L). (c) ALT test before implantation, at 1 month and 4 months after implantation. The threshold value was 5 times the upper limit of the normal level (50 U/L). (d) AST test before implantation, at 1 month and 4 months after implantation. The threshold value was 5 times the upper limit of the normal level (40 U/L). [file 12885_2021_8680_MOESM1_ESM.jpg]
